# Changing patterns of SARS-CoV-2 infection through Delta and Omicron waves by vaccination status, previous infection and neighbourhood deprivation: a cohort analysis of 2.7 M people

**DOI:** 10.1186/s12879-022-07878-2

**Published:** 2022-11-26

**Authors:** Mark A. Green, Daniel J. Hungerford, David M. Hughes, Marta Garcia-Fiñana, Lance Turtle, Christopher Cheyne, Matthew Ashton, Gary Leeming, Malcolm G. Semple, Alex Singleton, Iain Buchan

**Affiliations:** 1 Department of Geography and Planning, University of Liverpool, Liverpool, UK; 2 Centre for Global Vaccine Research, Institute of Infection, Veterinary and Ecological Sciences, University of Liverpool, Liverpool, UK; 3 NIHR Health Protection Research Unit in Gastrointestinal Infections, University of Liverpool, Liverpool, L69 7BE UK; 4 NIHR Health Protection Research Unit in Emerging and Zoonotic Infections, University of Liverpool, Liverpool, L69 7BE UK; 5 Department of Health Data Science, University of Liverpool, Liverpool, UK; 6 Public Health, Liverpool Local Authority, Liverpool, UK; 7 Civic Data Cooperative, University of Liverpool, Liverpool, UK; 8 Department of Public Health and Policy, University of Liverpool, Liverpool, UK

**Keywords:** COVID-19, Inequality, Vaccination, SARS-CoV-2

## Abstract

**Background:**

Our study examines if SARS-CoV-2 infections varied by vaccination status, if an individual had previously tested positive and by neighbourhood socioeconomic deprivation across the Delta and Omicron epidemic waves of SARS-CoV-2.

**Methods:**

Population cohort study using electronic health records for 2.7 M residents in Cheshire and Merseyside, England (3rd June 2021 to 1st March 2022). Our outcome variable was registered positive test for SARS-CoV-2. Explanatory variables were vaccination status, previous registered positive test and neighbourhood socioeconomic deprivation. Cox regression models were used to analyse associations.

**Results:**

Originally higher SARS-CoV-2 rates in the most socioeconomically deprived neighbourhoods changed to being higher in the least deprived neighbourhoods from the 1st September 2021, and were inconsistent during the Omicron wave. Individuals who were fully vaccinated (two doses) were associated with fewer registered positive tests (e.g., individuals engaged in testing between 1st September and 27th November 2021—Hazards Ratio (HR) = 0.48, 95% Confidence Intervals (CIs) = 0.47–0.50. Individuals with a previous registered positive test were also less likely to have a registered positive test (e.g., individuals engaged in testing between 1st September and 27th November 2021—HR = 0.16, 95% CIs = 0.15–0.18. However, the Omicron period saw smaller effect sizes for both vaccination status and previous registered positive test.

**Conclusions:**

Changing patterns of SARS-CoV-2 infections during the Delta and Omicron waves reveals a dynamic pandemic that continues to affect diverse communities in sometimes unexpected ways.

**Supplementary Information:**

The online version contains supplementary material available at 10.1186/s12879-022-07878-2.

## Background

Vaccination is the cornerstone of preventing severe COVID-19 disease among individuals infected with the SARS-CoV-2 virus [1]. Vaccines have also provided some protection from becoming infected with SARS-CoV-2, as has prior infection [2–4]. Unvaccinated individuals are at higher risk of severe illness, hospitalisation or death from COVID-19 [4, 5]. There is a lack of evidence over how long either vaccine- or infection-acquired immunity to SARS-CoV-2 infection and COVID-19 disease may last for [6]. Concerns over waning immunity [7, 8], and immune escape with the Omicron variant [9], led to the introduction of ‘booster’ vaccination programmes in late 2021 [10]. In addition to loss of biological protection, the risk behaviours of individuals change over time and may be influenced by feeling protected by vaccination or prior infection for longer than they actually are [11, 12]. Modelling of seasonal influenza vaccination programmes suggests that such behaviour changes can offset the effectiveness of vaccination programmes [13].

The COVID-19 pandemic has reinforced and amplified existing social inequalities in health. The number of infections, hospitalisations and deaths due to COVID-19 were disproportionally higher among residents of socioeconomically deprived neighbourhoods [14–16]. Vaccination uptake was also lower among deprived populations [17]. Assessing the importance of vaccine- and infection-acquired immunity are therefore social issues. However, current debates and evaluations of these issues largely ignore this social dimension. For example, estimates of vaccine effectiveness at reducing infections often present only unadjusted associations [18], which does not account for the differing levels of exposure to SARS-CoV-2 and vaccine uptake among different population and social groups.

The aim of this study is to examine if SARS-CoV-2 infections in England varied by vaccination status, if an individual had previously tested positive and by neighbourhood socioeconomic deprivation. We compare experiences during the epidemic curves of two SARS-CoV-2 variants: Delta and Omicron. The periods where these variants dominate infections represent an interesting case study due to high number of infections, high vaccine uptake, limited non-pharmaceutical interventions and changing public responses to national COVID-19 measures. It is also a period where all residents of England had access to free SARS-CoV-2 testing, allowing us to leverage electronic health records linked to testing records.

## Methods

### Data source

Data were accessed from the Combined Intelligence for Population Health Action (CIPHA; www.cipha.nhs.uk) resource. CIPHA is a population health management data resource set up to support responses to COVID-19. It constitutes linked electronic health records from routinely collected administrative data. Here, we used the population spine for CIPHA (all people registered with a GP and their primary care records), linked to NHS vaccination records and all registered SARS-CoV-2 tests.

### Study population

CIPHA contains linked pseudonymised electronic health care records for 2,864,997 people. We included people (n = 2,767,027) with a complete address who were resident during the study period in the integrated care region the CIPHA resource was set up to serve (Cheshire and Merseyside, England). Participants with missing data (n = 101) were excluded from analyses (other than missing data for ethnicity which we adjust for). For each period of analysis, we only include people who were alive up to the end of the period to minimise issues with immortal time bias.

### Study design

We selected three time periods to analyse:

1. *Delta—3rd June to 1st September 2021* We defined the start of the period as when Public Health England (now UK Health Security Agency) stated that the Delta variant was 99% of all infections [19].
2. *Delta—1st September to 27th November 2021* We selected this period to cover the wave of infections associated with the new school year (starting 1st September 2021) up to where the first case of Omicron was detected in England. The latter period was selected to focus our analyses on cases relating primarily to the Delta variant of SARS-CoV-2 to avoid any differences in risk of further infection or vaccine escape the Omicron variant may have.
3. *Omicron—13th December 2021 to 1st March 2022* We defined the start of this period as when sequencing data suggested that most positive tests were for Omicron. The period is then up to the end of available data at the time of analysis.

### Outcome variable

The primary outcome variable was time to SARS-CoV-2 infection (registered positive test) during each period. Time was defined as when the test was taken rather than when it was processed. Positive cases are compiled from data feeds supplied by the UK Health Security Agency, who share all Pillar 1 (tests in care settings) and Pillar 2 (tests in the community) positive tests which are linked within CIPHA. Positive cases are identified using both lateral flow and polymerase chain reaction (PCR) tests.

### Explanatory variables

We focused on three key explanatory variables: COVID-19 vaccination status, previous SARS-CoV-2 infection and neighbourhood socioeconomic deprivation.

Vaccination status was defined as the number of doses (of any vaccine type combination e.g. BNT162b2 (Pfizer-BioNTech) and the ChAdOx1 nCoV-19 (Oxford-AstraZeneca)) an individual had received (0–3). We identified the number of first doses received two weeks before the start of each period, and one week prior for two or three doses, which we define as the time to receive immune protection (following other research [3, 8]). The measure was then updated (i.e., time-varying) over time to account for people who received an additional vaccination during each study period. This was achieved using established methods through updating the time interval based on vaccination status, holding other covariates constant [20].

Previous SARS-CoV-2 infection (binary) was defined as whether an individual had a registered positive test from the start of the pandemic up to two weeks before the start of each period [21]. The measure was held constant and not time varying. We defined this two-week period as the time to develop immune protection. Infections were selected based on the first positive test, and subsequent positive tests occurring more than 90 days apart (which we defined as a further/subsequent infections). This definition follows established research elsewhere [8, 21]. We evaluated if this definition affected our results by introducing immortal time bias (i.e., some individuals could not test positive for parts of the study periods if they tested positive closer to the start period) through only including individuals who had a previous positive test at least 90 days before the start of the period as a sensitivity analysis.

Neighbourhood socioeconomic deprivation was measured through matching individual’s residence to the 2019 Index of Multiple Deprivation (IMD) [22]. The IMD is a multi-dimensional index of neighbourhood deprivation, based on seven weighted domains including income, employment, education and health. The IMD score is measured for Lower Super Output Areas (LSOAs) which are small zones representing neighbourhoods (~ 1500 people). Larger scores represent higher levels of socioeconomic deprivation. We also reported analyses by IMD decile to aid interpretation.

### Control variables

We accounted for demographic factors sex (male or female) and age. Age was included as a categorical variable to account for non-linear dynamics and produced a better fitting model than a continuous measure. Age is an important factor for different risks in exposure to SARS-CoV-2, as well as to reflect that the vaccination programme was rolled out by age group. Ethnicity was included to account for inequalities in both exposure to SARS-CoV-2 and vaccination uptake. Broad ethnic groups were used: White, Asian, Black, Mixed and Other. We also include ‘prefer not to say / missing’ as a category in our models, since they accounted for a large proportion of records and this can account for any issues with this group being different in causal behaviours. Health status was included to account for differences in behaviours, where people with long-term health conditions may ‘shield’ or minimise social contacts. We define health status (comorbidity) as if individuals had a registered Expanded Diagnosis Clusters codes (yes or no). Codes represent diseases, symptoms or conditions that are treated in ambulatory and inpatient hospital settings. Finally, we also adjusted for differences in testing dynamics by accounting for whether an individual had registered a negative test in the previous month.

### Statistical analyses

We found evidence of inequalities in registered test behaviours (Additional file 1: Table A). To minimise this potential bias in our regression analyses, we focused our analyses on two cohorts. First, we selected only individuals who reported a negative test in the month prior to each time period as a proxy for being engaged in testing. This is similar to a ‘test-negative’ study design which have been used for studying vaccine effectiveness [23]. Second, we analysed individuals who had received an influenza vaccine within a year of each time period as a proxy of being engaged in healthcare (i.e., likely to register a test even if unvaccinated and not disengaged with health care) [24]. For the Omicron period, we extended this time frame to 1^st^ September 2020 to fully capture the previous year’s influenza vaccination campaign. While our main models use all individuals, in a sensitivity analysis we restricted this population to just people aged 65 years and over as they are the focus of the UK influenza vaccination programme. Matching methods were also investigated for balancing populations across our exposure variables, but did not significantly alter the models and are not discussed here. We also reported analyses for all residents of Cheshire and Merseyside as a sensitivity analysis.

Descriptive statistics and visualisations were produced to summarise our data and identify key trends. Cox regression models were then used to predict the associations between our explanatory and control variables to our outcome variable (time to registered positive test). Hazard ratios and 95% confidence intervals were estimated from these models to summarise associations. Interaction effects for vaccine status and previous infection were tested, but not included in the results since they did not improve the model fit. We also stratified analyses by 10-year age group. This was to capture dynamics between children/adolescents and adults which will each have different modes of transmissions, risks and vaccination access [25]. We tested the proportional hazards assumption of models through estimating Schoenfeld residuals. Most associations met the proportional hazards assumption. Where the assumption was violated, estimates were not extreme and/or resulting plots did not display obvious violations suggesting that findings were potentially exaggerated by our large sample sizes. Alternative model specifications did not produce significantly different findings.

#### Patient and public involvement

No patients and the public were involved in this piece of research.

## Results

**Fig. 1:**
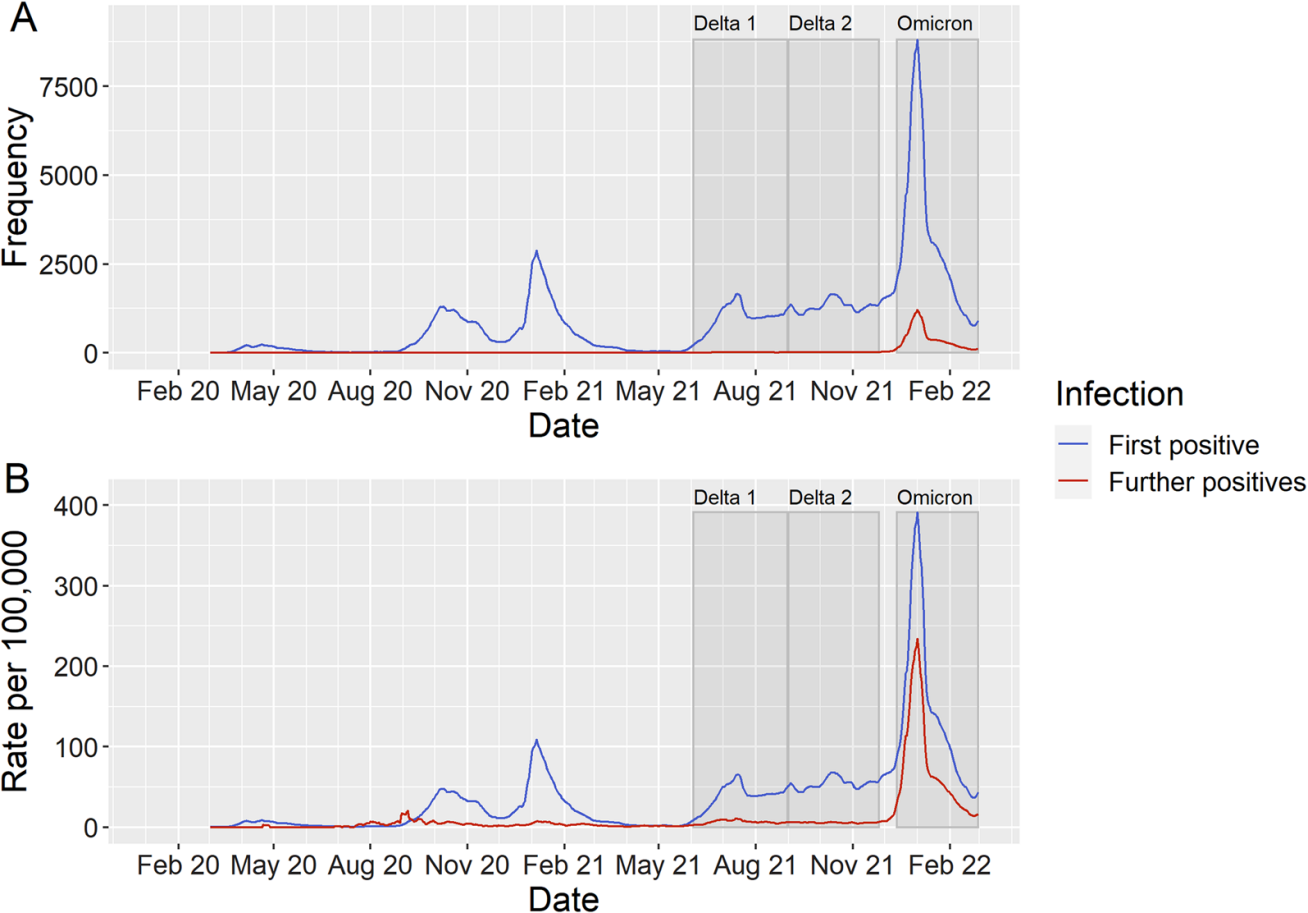
Seven day moving average for registered positive tests for all residents in Cheshire and Merseyside (England) by whether it was an individual’s first registered positive test (new infection) or further/subsequent positive test. **A** = Total number of cases, **B** = Total number of cases per 100,000 population

**Fig. 2:**
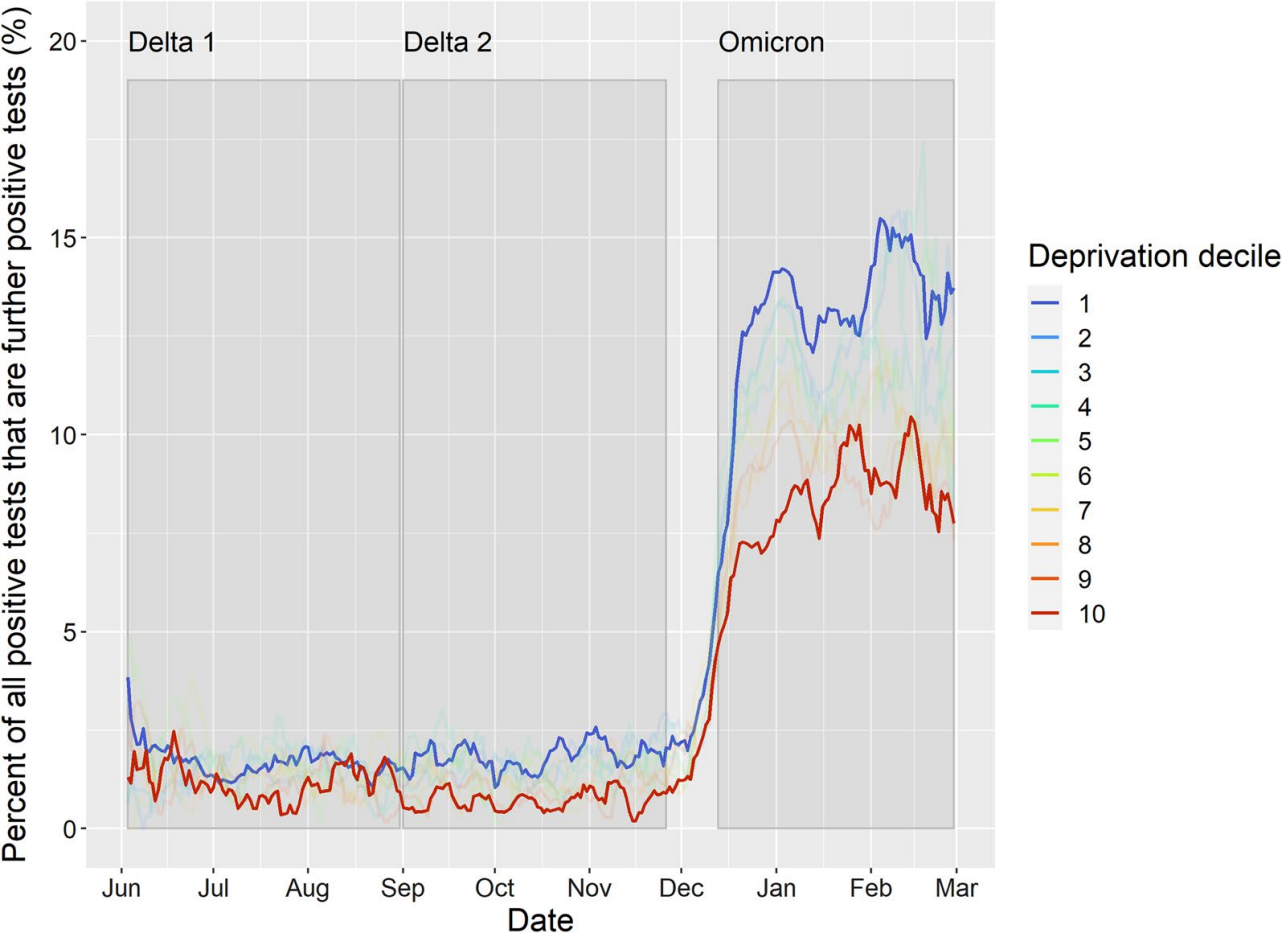
Seven day moving average for the percentage of all registered positive tests that were identified as a subsequent infection (≥ 2nd positive registered test more than 90 days apart) by decile of deprivation (3rd June 2021–2nd February 2022). Note: 1 = most deprived decile, 10 = least deprived decile, other deciles set to low transparency to minimise distraction

Table 1 presents the descriptive characteristics of our cohort. Figure 1 presents trends in registered positive cases for all residents since the start of the pandemic to contextualise our three periods. The number of cases was high during both Delta periods compared to previous waves. Omicron saw large growth in cases (10.5% of all residents registered a positive test; more than twice as high as both Delta periods) from new infections and the emergence of subsequent infections that almost reach the levels of new infections during the two Delta periods. We estimated that 11.4% of positive tests during the Omicron period were subsequent positive tests (in the other two periods, this figure was < 1%). In particular, incidence of further infections were roughly twice as high in the most deprived compared to least deprived areas (Fig. 2). Percentage of people with registered positive tests across our exposure variables are described in Additional file 1: Table B.

**Table 1:** Sample characteristics for each period. Note: Values are frequency counts (percentage) unless specified

	Delta (3rd June–1st Sept 2021)			Delta (1st Sept–27th Nov 2021)			Omicron (13th Dec–2nd Feb 2022)		
	All residents	Negative test	Influenza vaccinated	All residents	Negative test	Influenza vaccinated	All residents	Negative test	Influenza vaccinated
Number of individuals [n]	2,722,708	321,676	937,054	2,716,029	376,864	932,416	2,708,637	445,427	906,193
Registered positive test	3.4%	4.6%	2.2%	4.3%	5.7%	4.4%	10.5%	17.4%	9.7%
Explanatory variables									
Vaccination status									
Unvaccinated	47.2%	35.7%	23.5%	36.0%	21.1%	22.0%	31.8%	19.7%	22.4%
1 dose	16.2%	18.1%	9.8%	6.5%	7.6%	1.2%	5.3%	5.6%	3.7%
2 doses	36.5%	46.0%	66.4%	57.3%	71.0%	76.5%	32.8%	35.9%	16.4%
3 doses	0.2%	0.3%	0.2%	0.2%	0.4%	0.3%	30.1%	38.9%	57.5%
Previous infection	6.4%	9.4%	5.4%	9.2%	13.1%	7.1%	14.0%	16.1%	12.6%
Deprivation Score [mean (sd)]	28.8 (20.9)	25.7 (19.6)	25.1 (19.7)	28.8 (20.9)	25.7 (19.7)	25.0 (19.7)	28.8 (20.9)	25.2 (19.5)	25.1 (19.7)
Covariates									
Age [mean (sd)]	41.7 (23.5)	43.1 (22.4)	53.1 (26.7)	41.6 (23.5)	43.8 (21.0)	53.0 (26.6)	41.5 (23.4)	43.0 (22.0)	50.5 (27.5)
Sex									
Female	50.2%	59.2%	54.3%	50.2%	57.0%	54.4%	50.2%	57.1%	54.3%
Male	49.8%	40.8%	45.7%	49.8%	43.0%	45.6%	49.8%	42.9%	45.7%
Ethnic group									
White	73.2%	78.4%	82.8%	73.2%	77.1%	82.7%	73.1%	77.5%	82.0%
Asian or Asian British	1.4%	1.0%	1.0%	1.4%	1.0%	1.0%	1.4%	1.1%	1.1%
Black or Black British	0.7%	0.6%	0.5%	0.7%	0.6%	0.5%	0.7%	0.6%	0.5%
Mixed ethnicity	1.7%	1.7%	1.3%	1.7%	1.6%	1.3%	1.7%	1.6%	1.4%
Prefer not to say/Missing	8.2%	5.0%	2.6%	8.2%	6.0%	2.6%	8.2%	5.8%	2.7%
Other ethnicity	14.8%	13.3%	11.9%	14.8%	13.6%	11.9%	14.8%	13.4%	12.4%
Registered health issue	51.1%	59.3%	64.8%	51.0%	56.6%	64.7%	51.0%	57.7%	63.4%

Values are percentages unless specified. Characteristics are calculated at baseline for each time period

Tables 2 (individuals engaged in testing) and 3 (individuals engaged in healthcare) presents findings from a series of Cox regression models predicting factors associated with time to registered positive test. There was agreement in associations across both model types for the two Delta periods, with less consistent findings for the Omicron period.

**Table 2:** Results for a Cox regression predicting time to a registered positive test for individuals who had registered a negative lateral flow test within a month of the time period start date (as a proxy for testing engaged)

	Delta (3rd June–1st Sept 2021)			Delta (1st Sept–27th Nov 2021)			Omicron (13th Dec–2nd Feb 2022)		
	HR	LCI	UCI	HR	LCI	UCI	HR	LCI	UCI
Unvaccinated	Reference			Reference			Reference		
Unadjusted									
1 dose	0.70	0.66	0.74	0.47	0.44	0.51	1.16	1.11	1.22
2 doses	0.40	0.39	0.42	0.48	0.47	0.50	1.66	1.61	1.70
3 doses	0.41	0.28	0.60	0.24	0.19	0.30	0.86	0.84	0.89
Previous infection	0.25	0.22	0.27	0.16	0.15	0.18	0.73	0.71	0.76
Deprivation Score	1.005	1.004	1.006	0.995	0.994	0.996	1.004	1.004	1.005
Adjusted*									
1 dose	0.64	0.60	0.69	0.68	0.63	0.73	1.04	0.99	1.10
2 doses	0.53	0.51	0.56	0.66	0.63	0.68	1.30	1.26	1.34
3 doses	0.52	0.35	0.76	0.33	0.26	0.41	0.93	0.90	0.96
Previous infection	0.23	0.21	0.26	0.15	0.14	0.17	0.61	0.59	0.63
Deprivation Score	1.002	1.001	1.003	0.996	0.995	0.997	1.002	1.001	1.002

Definitions: HR = Hazard Ratio, LCI = 95% Lower Confidence Interval, UCI = 95% Upper Confidence Interval

Deprivation score is numerical, with increasing values representing higher levels of deprivation

*Adjusted for age (10-year age bands), sex, ethnicity, long-term illness, time varying vaccination status (with an interaction to time), previous infection status (and interaction to time), and 2019 Index of Multiple Deprivation score

Unadjusted associations for both Delta waves showed that people who were vaccinated had lower likelihoods of registered positive test for SARS-CoV-2. For instance, in individuals engaged in testing we estimated that people who were fully vaccinated (2 doses) were 60% (Hazard Ratio (HR) = 0.40, 95% Confidence Intervals (CIs) = 0.39–0.42) and 52% (HR = 0.48, 95% CIs = 0.47–0.50) less likely to have a registered positive test in the first and second Delta waves respectively compared to unvaccinated people. In individuals engaged in healthcare, we estimated a larger effect size with individuals who were fully vaccinated being 63% (HR = 0.37, 95% CIs = 0.36–0.39) and 66% (HR = 0.34, 95% CIs = 0.33–0.34) less likely to have a registered positive test in the first and second Delta waves respectively compared to unvaccinated people. After adjusting for other demographic and social factors that may affect exposure to the virus, the strength of associations reduced but remained negatively associated (i.e., HR < 1 and 95% CIs did not cross 1). In the second Delta wave (1st September to 27th November 2021), we observed a stronger protective effect in people who had received 3 doses in both models (i.e., fully vaccinated and ‘boosted’).

Associations during the Omicron period were different to the previous Delta periods and varied between models. For both models, unadjusted associations suggested positive associations in one or two doses, and a negative association for three doses (both compared to unvaccinated populations). For example, individuals engaged in testing with three doses were 14% less likely (HR = 0.86, 95% CIs = 0.84–0.89) and individuals who were healthcare engaged were 23% less likely (HR = 0.77, 95% CIs = 0.75–0.78). In adjusted models, negative associations for three doses remained in both models although effect sizes were smaller. This suggests that following adjustment for known risk factors that may affect exposure to SARS-CoV-2, individuals who were boosted were less likely to have a registered positive test.

People with a previous registered positive test had lower likelihood of having a registered positive test in each period across both models. Unadjusted effect sizes were large. For example, between 1st September and 27th November 2021 (Delta) we estimated that individuals with had a previous registered positive test were 84% (testing engaged model HR = 0.16, 95% CIs = 0.15–0.18; Table 2) and 86% (healthcare engaged model HR = 0.14, 95% CIs = 0.13–0.16; Table 3) less likely to have tested positive than compared to those who had not. Associations were consistent following adjusting for other covariates. The unadjusted effect size was smaller in the Omicron period (testing engaged model HR = 0.73, 95% CIs = 0.71–0.76; healthcare engaged model HR = 0.58, 95% CIs = 0.56–0.60), although effect sizes strengthened upon adjustment. Sensitivity analyses suggested that these associations remained consistent following assessing if our measure was affected by immortal time bias (Additional file 1: Table C).

**Table 3:** Full model results for a Cox regression predicting time to a registered positive test for individuals who had received an influenza vaccination within a year of the time period start date (as a proxy for healthcare engaged)

	Delta (3rd June–1st Sept 2021)			Delta (1st Sept–27th Nov 2021)			Omicron (13th Dec–2nd Feb 2022)		
	HR	LCI	UCI	HR	LCI	UCI	HR	LCI	UCI
Unvaccinated	Reference			Reference			Reference		
Unadjusted									
1 dose	0.69	0.65	0.74	0.36	0.33	0.40	1.27	1.21	1.32
2 doses	0.37	0.36	0.39	0.34	0.33	0.34	1.39	1.35	1.43
3 doses	0.43	0.32	0.59	0.13	0.11	0.15	0.77	0.75	0.78
Previous infection	0.19	0.17	0.22	0.14	0.13	0.16	0.58	0.56	0.60
Deprivation Score	1.0061	1.0054	1.0067	0.9951	0.9946	0.9956	1.0009	1.0005	1.0013
Adjusted*									
1 dose	0.66	0.61	0.72	0.53	0.47	0.60	0.98	0.93	1.03
2 doses	0.55	0.51	0.59	0.60	0.57	0.64	1.15	1.09	1.21
3 doses	0.63	0.46	0.86	0.28	0.24	0.34	0.94	0.89	0.99
Previous infection	0.17	0.15	0.19	0.11	0.10	0.13	0.45	0.44	0.47
Deprivation Score	1.0031	1.0024	1.0038	0.9925	0.9920	0.9930	0.9992	0.9987	0.9996

Definitions: HR = Hazard Ratio, LCI = 95% Lower Confidence Interval, UCI = 95% Upper Confidence Interval

Deprivation score is numerical, with increasing values representing higher levels of deprivation

*Adjusted for age (10-year age bands), sex, ethnicity, long-term illness, number of tests in previous month, time varying vaccination status (with an interaction to time), previous infection status (and interaction to time), and 2019 Index of Multiple Deprivation score

The associations for neighbourhood deprivation vary across each time period. In the first period (Delta—3rd June to 1st September 2021), we estimated positive associations in both models indicating that individuals in more deprived areas were more likely to have a registered positive test. To aid interpretation of this effect, we also estimated a model using national decile of deprivation (Additional file 1: Tables D and E). Individuals engaged in testing who resided in the least deprived decile were 24% less likely (HR = 0.76, 95% CIs = 0.72–0.81) and individuals who were healthcare engaged were 33% less likely (HR = 0.67, 95% CIs = 0.63–0.70), both compared to people in the most deprived decile.

In the second Delta period (1st September to 27th November 2021), the direction of the association was negative suggesting that as areas become more deprived, registered positive tests decreased. Individuals engaged in testing who resided in the least deprived decile were 37% more likely (HR = 1.37, 95% CIs = 1.30–1.44) and individuals who were healthcare engaged were 37% more likely (HR = 1.37, 95% CIs = 1.32–1.42), both compared to people in the most deprived decile (Additional file 1: Tables D and E). Age-stratified models suggest that the reversal of social inequalities appears to be driven by cases in children and older adults (Additional file 1: Figure A).

**Fig. 3:**
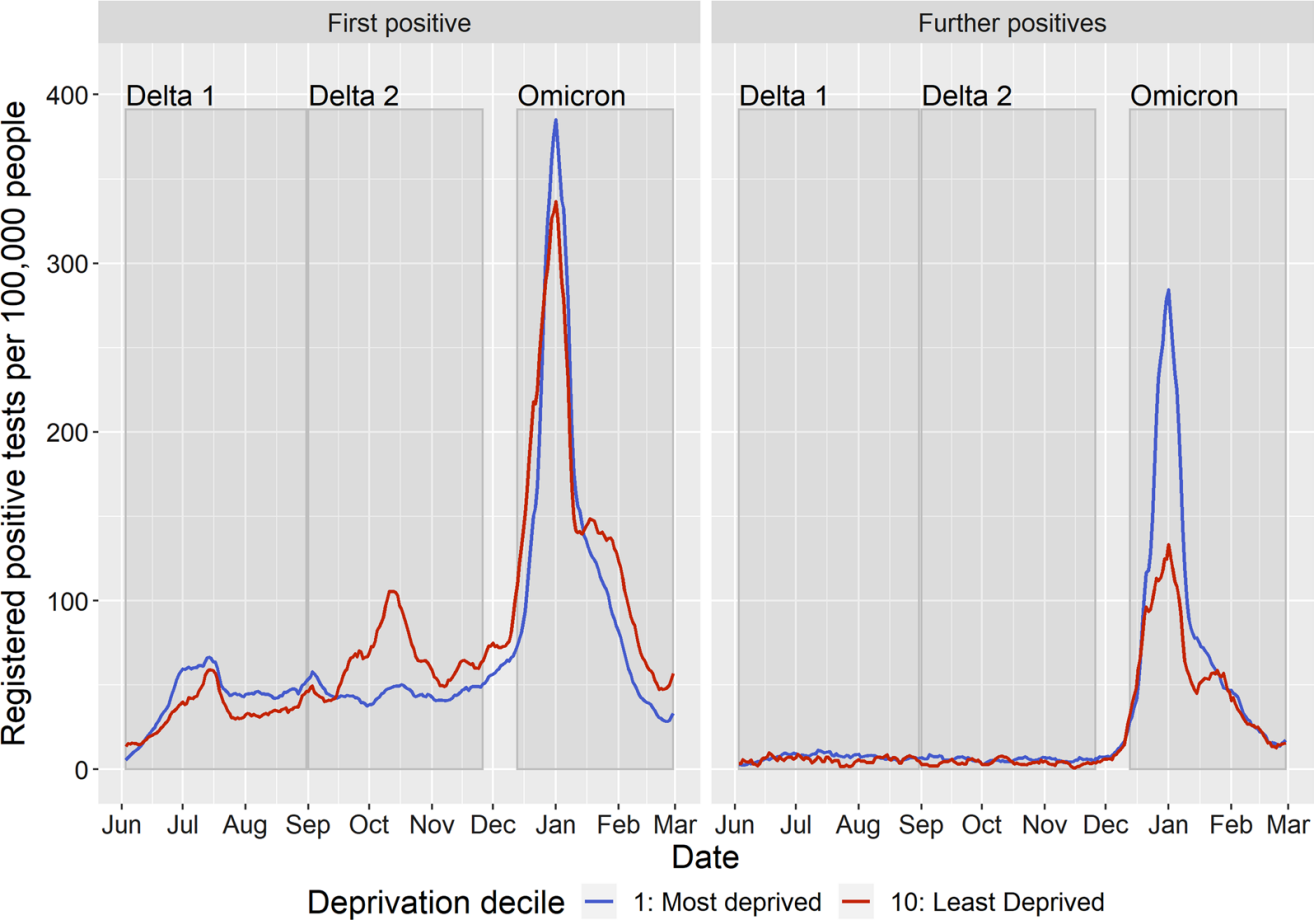
Comparison of seven day moving average for the number of residents in Cheshire and Merseyside per 100,000 people who registered a COVID-19 positive test for the most and least deprived deciles by whether it was an individual’s first registered positive test or a further/subsequent infection (3rd June 2021–2nd February 2022)

In the Omicron period (13th December 2021 to 28th February 2022), associations for deprivation showed diverging patterns across our models. Associations were positive in the testing engaged model (Table 2) and negative following adjustment in the healthcare engaged model (Table 3). This reflects the complexity in identifying associations over this period, where deprived and less deprived communities had the highest rates of registered positive tests at different points (Fig. 3). Initially incidence rates were higher in the least deprived decile, with trends reversing due to a larger peak of infections in the most deprived decile post-Christmas. By the end of the period, social inequalities had reversed again with more positive tests in the least deprived decile. For subsequent infections, the social gradient is more distinct with higher rates in the most deprived decile for most of the period before converging together (Fig. 2).

Our results were broadly consistent when analysing all residents in Cheshire and Merseyside (Additional file 1: Table F). The only exception was for vaccination status in the Omicron period, where we found positive associations for all vaccination doses (although any interpretation should be made cautiously due to the level of bias in these data). Similarly, our results were broadly consistent when restricting the healthcare engaged individuals to only people aged 65 years and over (Additional file 1: Table G).

## Discussion

### Key results

Our study details the complex changes over time in who was affected by the COVID-19 pandemic. While number of cases were high during the Delta waves, Omicron saw unprecedented numbers of cases with 10.5% of people in Cheshire and Merseyside having a registered positive test. Subsequent infections were identified in 11% of these tests, with rates higher in deprived areas. The types of people with registered positive tests has changed widely. Initially, social inequalities were evident with registered positive tests higher in the most deprived areas. Since 1st September 2021, this has been less consistent with more registered positive tests in the least deprived areas (partly driven by patterns in children and adolescents). While there were fewer registered positive tests in vaccinated populations, this changed with Omicron. Finally, we find that people with a previous registered positive test were far less likely to have a subsequent registered positive test.

### Interpretation

Our study does not assess vaccine effectiveness or vaccine impact on SARS-CoV-2 infection or COVID-19 disease. Rather it describes the types of people with registered positive tests for SARS-CoV-2 during the Delta and Omicron waves, and the complexity in being able to tease out these associations. Our analyses demonstrated that most new infections in the Delta wave occurred among unvaccinated populations. This association, however, becomes less clear with the emergence of Omicron where in individuals engaged in testing we observe more registered positive tests in individuals who were fully vaccinated (but not for those who were boosted or in individuals engaged with healthcare). This is not to suggest that being vaccinated places people at greater risk of being infected. Causal explanations for this association may include behavioural changes, such as increased physical contacts and working outside the home following vaccination increasing exposure to the virus [11, 12]. Evidence in England suggests that while individuals did not change behaviours after being vaccinated, increasing population vaccination levels were associated to changes in risk-compensatory behaviours and social contacts [26]. In addition, as most people get vaccinated or infected, the pool of unvaccinated people most susceptible to infection becomes smaller. It is plausible that this group is very different behaviourally and socially, and aversion to vaccination may translate to aversion to receiving or registering a test. For example, in the SIREN study where they test all individuals, they find fewer infections in vaccinated groups for each of our study periods [18], although the same study also has showed waning protection of vaccines in line with our findings [8]. Additionally, their analyses do not adjust for other covariates that may explain exposure to the virus. Finally, evidence has suggested that vaccines may have offered less protection to the Omicron variant due to immune escape [9, 27].

We find fewer registered positive tests in individuals with a previous positive test, with estimated effect sizes relatively larger than compared to vaccination status. This effect remains following adjusting demographic and social characteristics. The under-reporting of tests in individuals with a previous registered positive test may partly explain this difference. Immunity responses may also be different between vaccines and natural infections [6]. A large protective effect in natural infection has been reported elsewhere [8, 21]. Our estimated effect size reduced during the Omicron period, suggesting that the variant may be more effective at immune escape when compared to Delta. This is further highlighted by the larger percentage of subsequent infections identified.

Our findings should not be interpreted as naturally acquired immunity being recommended over vaccination. It is difficult to fairly compare effects across different variable types to identify which is most important and our methods do not allow for this. The people in our study who were previously infected excludes those that died of COVID-19, and the benefits of safe and effective vaccines have been clearly demonstrated in reducing COVID-19 hospitalisations and deaths [1, 4, 5]. However, our analyses might give some clues as to why England has not witnessed ‘herd immunity’ despite high levels of vaccination uptake.

From 1st September 2021, we found evidence of SARS-CoV-2 infections being higher in the least socioeconomically deprived communities. This remains in contrast to trends earlier in the pandemic, which had seen consistently higher infections in the most deprived areas [15], although not always [16]. The reversal of the social gradient in the Delta wave may be explained by several factors. One explanation may be the large protective effect of previous infections that we found. When combined with the concentration of infections in deprived areas in previous waves [15], this may have logically led to reduced population susceptibility to infections in more deprived communities during the Delta wave. When Omicron arrives it ‘resets’ these patterns since it is effectively a new serotype with immune escape [28], and the most deprived areas are affected more again. However, our analyses suggest that the reversal of the gradient was independent of previous infection and vaccination status of communities. A second explanation may regard the heterogeneity of social networks. The increasing socioeconomic segregation of who lives where [29] and school intakes [30], combined with low socioeconomic mixing and contact [31], may produce waves of infections that do not transfer between social groups and their closed networks. Our age stratified models suggest the reversal of social inequalities was strongest in children and adolescents, suggesting the importance of school dynamics in driving infections during Delta and Omicron [25]. Finally, inequalities in testing dynamics may produce an artefactual effect. Lower propensity to get tested or to register a test in deprived areas may bias our observations [32].

### Limitations

There are limitations to our data source. CIPHA is based on all individuals registered with a GP. While this captures most people in the region, we do not have information for those individuals who are not registered which may introduce bias to our data. SARS-CoV-2 infections were identified based on a registered positive test. There was limited community testing availability during the first wave of infections and access to lateral flow tests were not available until late 2020 (6th November in Liverpool only, 3rd December rest of region). These issues may lead to missed infections that would not be reported in our data resulting in under-counts for previous positive tests. Not all individuals may get tested, nor register their test, leading to undercounts of infections in our measures. We do not know the extent of this under-reporting, including how it varies across our exposure variables, which may introduce selection bias. We attempted to account for some of these issues by restricting analyses to individuals who had registered a negative test in the month before due to established inequalities in testing uptake [32]. The impact of this can be seen by comparing the models to analyses for all residents (e.g., Table 2 and Additional file 1: Table F). We also report significant inequalities in who reported negative tests across our exposure variables (Additional file 1: Table A) which may bias underlying associations. The range of bias we are unable to observe shows how difficult it is to investigate these phenomena using routine data, so our results should not be over-interpreted.

Our analyses are descriptive and exploratory. We could not investigate the mechanisms that may underlie the associations we report (e.g., the processes that explain why social inequalities changed over time). We also are unable to account for all potential confounders or explanatory factors. For example, we did not have access to information matching individuals to households which may help to account for household transmission. It is plausible that our model adjustment may not be able to disentangle the association between demographic/social factors and our exposures (including risk behaviours and testing frequency). In particular, we only have access to area-level measures for socioeconomic deprivation which means any attempt to understand individual-level processes are at risk of the ecological fallacy. Future research should evaluate the potential reasons behind the relationships we describe, moving beyond the use of routine data to address some of the limitations of our analyses (e.g., social network analyses to study if the social segregation of communities explains inequalities flipping or using qualitative data to understand the contextual differences between poorer and affluent areas).

## Conclusion

Using linked NHS and public health testing records for 2.7 M people in Cheshire and Merseyside, our study reveals the dynamic nature of SARS-CoV-2 infections through the Delta and Omicron waves. Socially patterned immunity by vaccination and prior infection resulted in social flips in who is infected, producing complex pictures of socioeconomic inequalities. Finding ways to effectively communicate the risks in exposure and infections among populations based on the changing dynamics we uncovered remains important. In the context of ‘living with COVID-19’ and the removal of most non-pharmaceutical interventions, our findings suggest that highly infectious SARS-CoV-2 variants will continue to spread unequally through society but not always in expected ways.

## Supplementary Information

**Additional file 1:** Additional results and sensitivity analyses that supplement the core paper and are referred to in text.

## Data Availability

Data are accessible via CIPHA (https://www.cipha.nhs.uk/). Requests can be made to the Data Access Committee for extracts of the larger-scale data which cannot be released openly due to information governance requirements. All analyses were undertaking using open source R statistical software and code is made openly available here https://github.com/markagreen/social_flip_COVID-19.
