# Supplementary material for: Changing patterns of SARS-CoV-2 infection through Delta and Omicron waves by vaccination status, previous infection and neighbourhood deprivation: a cohort analysis of 2.7 M people
Source: BMC Infect Dis. 2022 Nov 26;22:889. doi: 10.1186/s12879-022-07878-2 (PMC9701390; doi:10.1186/s12879-022-07878-2)
Supplement: Supplementary file 1 — Additional file 1: Additional results and sensitivity analyses that supplement the core paper and are referred to in text. [file 12879_2022_7878_MOESM1_ESM.pdf]

Appendix

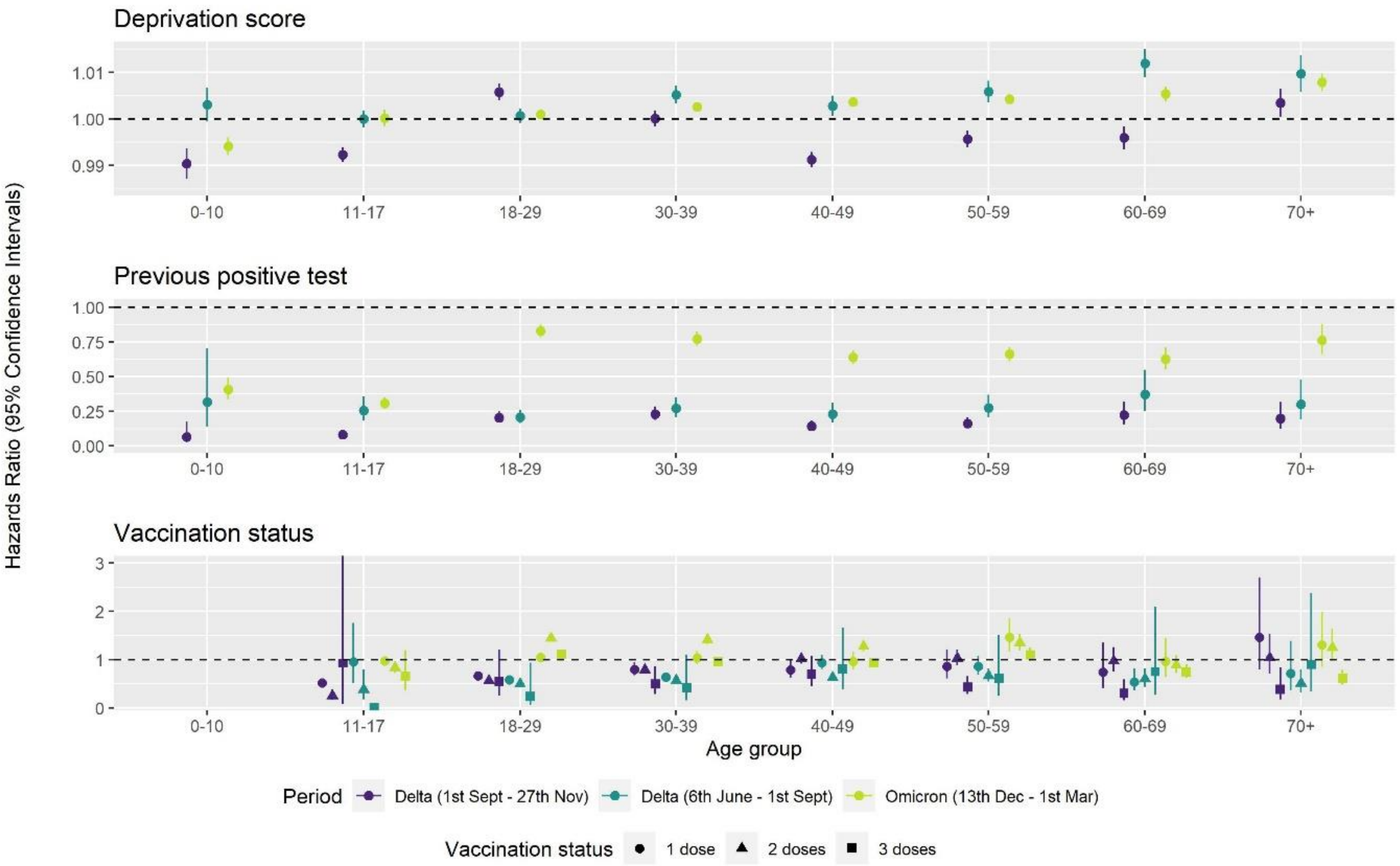

Figure A: Age-stratified Cox regression models predicting time to registered positive test. Note: Coefficients for vaccination status for children aged 0-10 were not included, since due to low numbers model fit was not possible. Models are unadjusted for other covariates (model adjustment does not significantly change patterns). Reference categories are no previous positive test for previous positive test, and no vaccination doses for vaccination status.

Table A: Percentage of all participants in Cheshire and Merseyside who registered a negative COVID-19 test during each study period (i.e., within each outcome period).

	Delta (3rd June - 1st Sept 2021)			Delta (1st Sept - 27th Nov 2021)			Omicron (13th Dec - 2nd Feb 2022)		
	All residents	Negative test	Influenza vaccinated	All residents	Negative test	Influenza vaccinated	All residents	Negative test	Influenza vaccinated
Unvaccinated	24.4	66.4	28.5	27.9	60.3	38.2	19.9	47.8	27.0
1 dose	31.1	73.8	33.3	33.1	60.9	34.4	41.3	69.5	57.2
2 doses	32.2	76.0	30.6	36.0	70.4	34.8	30.4	60.3	31.8
3 doses	40.9	84.0	38.3	44.3	77.8	42	34.3	72.2	33.1
No previous infection	27.7	71.7	29.8	32.1	67.2	34.9	27.4	62.2	31.3
Previous infection	38.1	77.6	40.2	41.1	69.9	43.9	37.4	67.1	40.3
Most deprived decile (1)	22.8	66.9	26.1	26.1	62.7	29.5	23.6	59.3	27.2
Decile 2	26.0	70.6	28.7	30.4	66.3	33.3	26.7	61.5	30.1
Decile 3	27.7	72.3	30.2	31.8	67.7	34.2	28.0	62.5	31.5
Decile 4	28.3	72.7	31.0	32.8	67.5	35.9	28.4	63.0	31.9
Decile 5	29.2	73.7	30.6	33.7	67.8	35.4	29.6	63.3	32.4
Decile 6	30.9	73.5	31.7	35.8	69.3	37.1	31.3	64.3	33.8
Decile 7	31.6	74.0	31.6	36.5	69.3	37.6	31.8	64.1	34.1
Decile 8	32.1	74.3	32.1	37.2	69.2	38.0	32.3	64.6	34.4
Decile 9	32.2	73.9	32.0	38.4	70.3	38.6	33.1	64.8	35.4
Least deprived decile (10)	34.1	76.2	33.6	40.1	70.9	40.5	34.5	65.8	37.1

Characteristics are calculated at baseline for each time period (and not time varying here).

Table B: Percentage of all participants with a registered positive test by exposure variables.

	Delta (3rd June - 1st Sept 2021)			Delta (1st Sept - 27th Nov 2021)			Omicron (13th Dec - 28th Feb 2022)		
	All residents	Negative test	Flu vaccinated	All residents	Negative test	Flu vaccinated	All residents	Negative test	Flu vaccinated
Vaccination status									
Unvaccinated	4.5	7.0	4.1	5.9	10.1	10.0	8.6	18.4	13.6
1 dose	3.1	4.0	2.4	2.7	3.6	2.9	12.6	18.8	14.6
2 doses	1.9	2.9	1.5	3.5	4.7	2.9	13.8	19.7	10.6
3 doses	2.5	3.0	1.8	3.9	4.3	3.4	8.6	14.7	7.5
Previous infection									
No	3.5	4.9	2.3	4.7	6.5	4.7	10.9	18.4	10.1
Yes	0.7	1.2	0.4	0.6	1.0	0.6	8.4	12.5	6.6
Deprivation level									
Most deprived decile (1)	3.6	5.3	2.7	3.6	4.9	3.8	9.9	18.8	9.4
Decile 2	3.5	4.9	2.4	4.2	5.4	4.4	10.7	18.5	9.9
Decile 3	3.4	4.6	2.3	4.3	5.6	4.4	10.9	17.9	10.1
Decile 4	3.5	4.9	2.5	4.2	5.7	4.5	10.7	18.5	10.0
Decile 5	3.4	4.4	2.2	4.3	5.5	4.3	10.7	17.8	9.6
Decile 6	3.1	4.1	2.0	4.6	6.0	4.6	10.7	16.7	9.6
Decile 7	3.1	4.3	2.0	4.6	5.9	4.3	10.6	16.6	9.6
Decile 8	3.2	4.3	2.1	4.7	5.9	4.6	10.7	16.4	9.5
Decile 9	3.0	3.9	1.9	5.0	6.5	5.0	10.8	16.3	9.5
Least deprived decile (10)	2.9	4.1	1.8	5.1	6.7	5.1	10.6	16.2	9.8

Note: Values are percentages. Characteristics are calculated at baseline for each time period.

Table C: Model results when redefining the measure for previous positive test (i.e., excluding all individuals with a registered positive test within 90 days of the start of the period) to assess the extent of immortal time bias.

	**Delta (3rd June - 1st Sept 2021)**			**Delta (1st Sept - 27th Nov 2021)**			**Omicron (13th Dec - 2nd Feb 2022)**		
	**HR**	**LCI**	**UCI**	**HR**	**LCI**	**UCI**	**HR**	**LCI**	**UCI**
Individuals who were registered a negative test									
Unadjusted									
Previous infection	0.23	0.21	0.26	0.19	0.17	0.21	0.87	0.85	0.90
*Adjusted**									
Unvaccinated	Reference			Reference			Reference		
1 dose	0.64	0.60	0.69	0.68	0.63	0.73	1.02	0.97	1.08
2 doses	0.53	0.51	0.56	0.65	0.62	0.68	1.29	1.25	1.34
3 doses	0.52	0.35	0.76	0.32	0.25	0.40	0.89	0.86	0.92
Previous infection	0.22	0.19	0.25	0.19	0.17	0.21	0.71	0.69	0.73
Deprivation Score	1.0020	1.0012	1.0028	0.9961	0.9954	0.9968	1.0011	1.0007	1.0016
Individuals who were flu vaccinated									
Unadjusted									
Previous infection	0.18	0.16	0.21	0.17	0.15	0.19	0.82	0.80	0.85
*Adjusted**									
Unvaccinated	Reference			Reference			Reference		
1 dose	0.66	0.61	0.72	0.52	0.46	0.59	0.93	0.88	0.98
2 doses	0.55	0.51	0.59	0.60	0.56	0.63	1.12	1.06	1.18
3 doses	0.63	0.46	0.86	0.28	0.23	0.33	0.89	0.84	0.93
Previous infection	0.16	0.14	0.18	0.15	0.14	0.17	0.63	0.61	0.65
Deprivation Score	1.0031	1.0024	1.0038	0.9925	0.9920	0.9930	0.9987	0.9983	0.9991

Definitions: HR = Hazard Ratio, LCI = 95% Lower Confidence Interval, UCI = 95% Upper Confidence Interval

Note: Deprivation score is numerical, with increasing values representing higher levels of deprivation

* Adjusted for age (10-year age bands), sex, ethnicity, long-term illness, number of tests in previous month, time varying vaccination status (with an interaction to time), previous infection status (and interaction to time), and 2019 Index of Multiple Deprivation score

Table D: Results for a Cox regression predicting time to a registered positive test by deprivation decile for individuals who had registered a negative lateral flow test within a month of the time period start date (as a proxy for testing engaged).

	Delta (3rd June - 1st Sept 2021)			Delta (1st Sept - 27th Nov 2021)			Omicron (13th Dec - 2nd Feb 2022)		
	**HR**	**LCI**	**UCI**	**HR**	**LCI**	**UCI**	**HR**	**LCI**	**UCI**
Unadjusted									
Most deprived (1)	Reference			Reference			Reference		
Decile 2	0.92	0.87	0.98	1.10	1.04	1.16	0.95	0.92	0.98
Decile 3	0.86	0.81	0.92	1.13	1.07	1.20	0.92	0.89	0.96
Decile 4	0.93	0.87	0.99	1.15	1.08	1.22	0.95	0.92	0.99
Decile 5	0.85	0.80	0.91	1.11	1.05	1.18	0.92	0.89	0.95
Decile 6	0.78	0.73	0.83	1.20	1.13	1.27	0.83	0.80	0.86
Decile 7	0.80	0.75	0.86	1.21	1.14	1.28	0.80	0.77	0.83
Decile 8	0.81	0.76	0.86	1.19	1.13	1.26	0.82	0.79	0.85
Decile 9	0.73	0.68	0.78	1.33	1.26	1.40	0.79	0.76	0.82
Least deprived (10)	0.76	0.72	0.81	1.37	1.30	1.44	0.73	0.71	0.76
*Adjusted**									
Most deprived (1)	Reference			Reference			Reference		
Decile 2	0.96	0.91	1.02	1.11	1.06	1.17	0.99	0.96	1.02
Decile 3	0.92	0.87	0.99	1.14	1.07	1.20	0.98	0.94	1.01
Decile 4	0.98	0.92	1.05	1.18	1.11	1.25	1.01	0.98	1.05
Decile 5	0.94	0.88	1.00	1.13	1.07	1.20	1.00	0.96	1.04
Decile 6	0.89	0.83	0.96	1.18	1.12	1.25	0.93	0.90	0.97
Decile 7	0.94	0.88	1.00	1.19	1.13	1.25	0.91	0.88	0.95
Decile 8	0.93	0.88	0.99	1.17	1.11	1.23	0.94	0.91	0.98

Decile 9	0.85	0.80	0.91	1.26	1.20	1.33	0.92	0.89	0.96
Least deprived (10)	0.89	0.84	0.95	1.27	1.21	1.33	0.88	0.85	0.91

Definitions: HR = Hazard Ratio, LCI = 95% Lower Confidence Interval, UCI = 95% Upper Confidence Interval

* Adjusted for age (10-year age bands), sex, ethnicity, long-term illness, number of tests in previous month, time varying vaccination status (with an interaction to time), previous infection status (and interaction to time).

Table E: Results for a Cox regression predicting time to a registered positive test by deprivation decile for individuals who had received a flu vaccination within a year of the time period start date (as a proxy for healthcare engaged).

	Delta (3rd June - 1st Sept 2021)			Delta (1st Sept - 27th Nov 2021)			Omicron (13th Dec - 2nd Feb2022)		
	**HR**	**LCI**	**UCI**	**HR**	**LCI**	**UCI**	**HR**	**LCI**	**UCI**
Unadjusted									
Most deprived (1)	Reference			Reference			Reference		
Decile 2	0.90	0.86	0.95	1.16	1.12	1.21	1.03	1.00	1.07
Decile 3	0.86	0.82	0.91	1.17	1.13	1.22	1.04	1.01	1.08
Decile 4	0.92	0.87	0.97	1.21	1.16	1.27	1.02	0.98	1.05
Decile 5	0.83	0.79	0.88	1.14	1.10	1.19	1.03	1.00	1.07
Decile 6	0.76	0.72	0.81	1.21	1.17	1.27	0.98	0.95	1.02
Decile 7	0.75	0.71	0.79	1.15	1.10	1.19	0.95	0.92	0.98
Decile 8	0.80	0.76	0.84	1.23	1.19	1.28	0.97	0.94	1.01
Decile 9	0.69	0.66	0.73	1.33	1.29	1.38	0.95	0.92	0.99
Least deprived (10)	0.67	0.63	0.70	1.37	1.32	1.42	0.92	0.89	0.95
*Adjusted**									
Most deprived (1)	Reference			Reference			Reference		
Decile 2	0.94	0.89	0.99	1.21	1.17	1.26	1.05	1.02	1.08
Decile 3	0.92	0.87	0.97	1.25	1.20	1.30	1.07	1.03	1.10
Decile 4	1.00	0.95	1.06	1.33	1.28	1.39	1.06	1.02	1.10
Decile 5	0.94	0.89	0.99	1.29	1.23	1.34	1.09	1.06	1.13
Decile 6	0.87	0.82	0.92	1.38	1.32	1.43	1.05	1.01	1.09

Decile 7	0.87	0.82	0.92	1.33	1.27	1.38	1.03	1.00	1.07
Decile 8	0.93	0.88	0.98	1.43	1.38	1.49	1.06	1.03	1.10
Decile 9	0.81	0.77	0.86	1.54	1.48	1.59	1.04	1.01	1.08
Least deprived (10)	0.78	0.74	0.82	1.54	1.49	1.60	1.01	0.97	1.04

Definitions: HR = Hazard Ratio, LCI = 95% Lower Confidence Interval, UCI = 95% Upper Confidence Interval

* Adjusted for age (10-year age bands), sex, ethnicity, long-term illness, number of tests in previous month, time varying vaccination status (with an interaction to time), previous infection status (and interaction to time).

Table F: Results for a Cox regression predicting time to a registered positive test for all residents in Cheshire and Merseyside.

	Delta (3rd June - 1st Sept 2021)			Delta (1st Sept - 27th Nov 2021)			Omicron (13th Dec - 2nd Feb 2022)		
	**HR**	**LCI**	**UCI**	**HR**	**LCI**	**UCI**	**HR**	**LCI**	**UCI**
Unadjusted									
Unvaccinated	Reference			Reference			Reference		
1 dose	0.88	0.86	0.90	0.63	0.61	0.65	1.70	1.66	1.73
2 doses	0.46	0.45	0.47	0.65	0.64	0.65	2.44	2.41	2.47
3 doses	0.54	0.45	0.65	0.34	0.30	0.39	1.13	1.12	1.15
Previous infection	0.23	0.22	0.25	0.15	0.14	0.15	0.81	0.79	0.82
Deprivation Score	1.0028	1.0025	1.0031	0.9939	0.9936	0.9941	0.9994	0.9991	0.9996
*Adjusted**									
Unvaccinated	Reference			Reference			Reference		
1 dose	0.86	0.84	0.88	0.90	0.87	0.93	1.42	1.39	1.46
2 doses	0.72	0.71	0.74	1.00	0.99	1.02	2.13	2.10	2.16
3 doses	0.76	0.63	0.92	0.60	0.53	0.67	1.61	1.58	1.64
Previous infection	0.20	0.18	0.21	0.13	0.12	0.13	0.63	0.62	0.64
Deprivation Score	0.9992	0.9989	0.9995	0.9927	0.9925	0.9930	0.9985	0.9983	0.9987

Definitions: HR = Hazard Ratio, LCI = 95% Lower Confidence Interval, UCI = 95% Upper Confidence Interval

Note: Deprivation score is numerical, with increasing values representing higher levels of deprivation

* Adjusted for age (10-year age bands), sex, ethnicity, long-term illness, number of tests in previous month, time varying vaccination status (with an interaction to time), previous infection status (and interaction to time), and 2019 Index of Multiple Deprivation score

Table G: Full model results for a Cox regression predicting time to a registered positive test for individuals who had received an influenza vaccination within a year of the time period start date and were aged 65 years and over (as a proxy for healthcare engaged).

	Delta (3rd June - 1st Sept 2021)			Delta (1st Sept - 27th Nov 2021)			Omicron (13th Dec - 2nd Feb 2022)		
	HR	LCI	UCI	HR	LCI	UCI	HR	LCI	UCI
Unadjusted									
Unvaccinated	Reference			Reference			Reference		
1 dose	0.36	0.24	0.54	1.02	0.62	1.67	1.22	1.13	1.33
2 doses	0.41	0.32	0.53	1.46	1.05	2.02	1.43	1.36	1.51
3 doses	0.50	0.29	0.86	0.40	0.26	0.61	0.76	0.73	0.79
Previous infection	0.27	0.20	0.36	0.15	0.11	0.20	0.61	0.57	0.65
Deprivation Score	1.0097	1.0083	1.0112	1.0011	0.9999	1.0022	1.0011	1.0003	1.0018
*Adjusted**									
Unvaccinated	Reference			Reference			Reference		
1 dose	0.35	0.23	0.52	1.05	0.64	1.71	0.97	0.89	1.07
2 doses	0.45	0.35	0.58	1.40	1.01	1.94	1.19	1.08	1.31
3 doses	0.57	0.33	0.97	0.42	0.27	0.64	0.96	0.87	1.06
Previous infection	0.23	0.17	0.31	0.12	0.09	0.16	0.47	0.44	0.50
Deprivation Score	1.0087	1.0072	1.0103	1.0009	0.9997	1.0020	0.9992	0.9984	1.0000

Definitions: HR = Hazard Ratio, LCI = 95% Lower Confidence Interval, UCI = 95% Upper Confidence Interval

Note: Deprivation score is numerical, with increasing values representing higher levels of deprivation

* Adjusted for age (10-year age bands), sex, ethnicity, long-term illness, number of tests in previous month, time varying vaccination status (with an interaction to time), previous infection status (and interaction to time), and 2019 Index of Multiple Deprivation score
